# Drugging the tumor microenvironment epigenome for therapeutic interventions in NSCLC

**DOI:** 10.7150/jca.111023

**Published:** 2025-02-18

**Authors:** Kostas A. Papavassiliou, Amalia A. Sofianidi, Vassiliki A. Gogou, Athanasios G. Papavassiliou

**Affiliations:** 1 First University Department of Respiratory Medicine, 'Sotiria' Chest Hospital, Medical School, National and Kapodistrian University of Athens, Athens, Greece.; 2 Department of Biological Chemistry, Medical School, National and Kapodistrian University of Athens, Athens, Greece.

For many years, cancer research was dominated by a tumor-centric perspective, where the focus was oriented exclusively on malignant cells, largely overlooking their neighboring environment. Recently, however, a paradigm shift has occurred, giving rise to a tumor microenvironment (TME)-centric view, which underscores the crucial role of the adjacent environment in driving the acquisition of cancer hallmarks 1. The TME is a dynamic and tumor-specific ecosystem, typically comprising malignant cells, surrounding fibroblasts-referred to as cancer-associated fibroblasts (CAFs)-endothelial cells, immune cells, as well as blood and lymph vessels 2. These components are engaged in intricate interactions fostering tumor initiation, development, and resistance to therapy. Notably, their tumor-promoting properties are enhanced by progressive epigenetic alterations, including histone modifications, DNA methylation, and chromatin remodeling, which collectively potentiate oncogenic transcription programs 3. Therefore, it is essential to adopt a more holistic approach in therapeutic strategies, reckoning with the epigenetic landscape of all components within the TME. The present commentary provides a synopsis of therapeutic interventions targeting the TME epigenome in non-small cell lung cancer (NSCLC), highlighting the latest advancements in this rapidly evolving field.

When considering strategies to target the TME epigenome in NSCLC, our primary focus is directed towards the cancer cells themselves. In this vein, histone deacetylase inhibitors (HDACi) (**Figure 1**) have shown promising antitumor activity, opening new avenues for NSCLC treatment. Clinically, the HDACi Vorinostat was evaluated in a phase I trial alongside the programmed cell death protein 1 (PD-1) inhibitor pembrolizumab, demonstrating enhanced survival outcomes in patients with advanced or metastatic NSCLC 4. Two other HDACi, Trichostatin A (originally developed as an antifungal agent) and Quisinostat (JNJ-2648158; an orally bioavailable second-generation HDACi), have proven effective in disrupting the integrity of the epithelial barrier in lung adenocarcinoma cells 5. With respect to ongoing clinical trials, the phase II trial NCT05141357 assesses the effectiveness of the benzamide HDACi Tucidinostat (HBI-8000) in conjunction with the PD-1 inhibitor nivolumab for treating advanced or metastatic NSCLC. Likewise, the phase II study NCT01928576 investigates the potential of epigenetic therapy using a combination of azacitidine, a DNA methyltransferase inhibitor (DNMTi) (**Figure 1**), and Entinostat, a synthetic benzamide derivative HDACi, administered together with nivolumab in patients with metastatic NSCLC. To explore the potential of azacitidine as a more effective regimen when administered directly into the lungs, a phase I/II clinical trial (NCT06694454) was launched to evaluate neoadjuvant inhaled azacitidine in combination with platinum-based chemotherapy and the programmed cell death-ligand 1 (PD-L1) durvalumab for operable early-stage NSCLC. This combined epigenetic-immunotherapy regimen paves the way for leveraging immune cells within the TME as therapeutic allies in NSCLC and is discussed in more detail below. Another ongoing phase II clinical trial (NCT04250246) evaluates guadecitabine, a novel DNMTi with improved pharmacokinetics causing genome-wide and nonspecific hypomethylation and inducing cell-cycle arrest at S-phase, alongside immune checkpoint blockage in patients with NSCLC and primary resistance to anti-PD-1/PD-L1 therapy. Decitabine (5-aza-2-deoxycytidine) is another DNMTi, which in preclinical studies suppressed NSCLC growth and reduced metastatic potential when combined with acetylsalicylic acid; this effect was mainly achieved via hindering the β-catenin-signal transducer and activator of transcription 3 (STAT3) signaling pathway 6. Furthermore, natural compounds exhibit significant potential for modulating the epigenetic landscape of cancer cells. Notable examples include curcumin (the active ingredient in turmeric plant), which exerts regulatory effects on non-coding RNA alterations 7, the traditional Chinese herbal medicine Jinfukang (JFK), which induces epigenetic modifications in the promoters of oncogenic genes 8, and cucurbitacin B (CuB; the most abundant and active member of cucurbitacins), which influences microRNA-related pathways 9. These compounds have demonstrated effectiveness in halting the progression of NSCLC cells 7-9. Nevertheless, considering their limited bioavailability, (nano)formulations providing ampler pharmacokinetic and pharmacodynamic profiles are currently being developed 10, 11.

CAFs have recently emerged as pivotal regulators of tumor development and progression. Their distinct epigenetic profiles contribute to NSCLC heterogeneity, being in part responsible for the failure of traditional NSCLC therapeutic modalities. Fimepinostat (CUDC-907), an inhibitor of both phosphoinositide 3-kinase (PI3K) and HDAC, has shown preclinical efficacy in hampering CAF cell proliferation and migration in NSCLC models 12. Additionally, given the abnormal involvement of the paracrine molecule transforming growth factor-beta (TGF-β) in CAF activation and epigenetic regulation, studies have explored pharmaceutical approaches to target this cytokine. In preclinical models, the selective TGF-β receptor I kinase inhibitor LY2109761 decreased the expansion of squamous cell carcinoma (SCC) CAFs *in vivo* 13*.* Regarding non-coding RNAs, a recent study underscored the therapeutic prospect of targeting a long non-coding RNA (lncRNA), LINC01614, to disrupt glutamine utilization and impede cancer progression in lung adenocarcinoma 14*.* Targeting transcription factor/co-factor complexes subjected to epigenetic modifications also presents a promising approach 15. The transcription factor suppressor of mothers against decapentaplegic homolog 3 (SMAD3) plays a decisive role in shaping the epigenetic profile of CAFs and its targeting with the potent and selective SMAD3 inhibitor SIS3 has demonstrated significant efficacy in preclinical lung cancer models 16.

Immune cells within the lung TME (tumor immune microenvironment, TIME) have the potential to play a vital anti-tumor role 17-20. When properly regulated, they can contribute to the fight against cancer cells by secreting a variety of cytokines, chemokines, and other soluble factors 21. Enhancer of Zeste homolog 2 (EZH2), a histone-lysine methyltransferase (HKMT) and the catalytic subunit of polycomb repressive complex 2 (PRC2) (**Figure 1**) is aberrantly involved in epigenetic gene silencing and serves as a critical regulator in the progression of various tumors. It has been demonstrated that EZH2 stimulates the expression of the C-C motif chemokine ligand 5 (CCL5; also known as RANTES), resulting in the recruitment of macrophages and facilitating lung cancer progression 22. Recent research indicates that disialoganglioside GD2 is aberrantly expressed in NSCLC, and its expression can be upregulated by the oral EZH2 inhibitor Tazemetostat, enhancing responsiveness to chimeric antigen receptor (CAR) T-cell therapy 23. Another oral, next-generation, dual EZH2/EZH1 inhibitor, Tulmimetostat (CPI-0209), is being evaluated in a phase Ib/II clinical trial (NCT05467748) alongside checkpoint (PD-1) blockade with pembrolizumab, enrolling patients with advanced NSCLC who have progressed under first or second-line treatment. Bromo- and extra-terminal domain (BET) family proteins are essential for chromatin remodeling and are engaged in transcriptional complexes regulated by epigenetic modifications 24. BET inhibition enhances antitumor immunity in a tumor necrosis factor (TNF)-dependent manner 24; the BET inhibitor ZEN003694 is currently being assessed in a phase II clinical trial (NCT05607108), accruing patients with SCC featuring a mutation in the HKMT-encoding *nuclear receptor-binding SET domain* (*NSD*)* protein 3* (*NSD3*) gene.

In summary, it is of utmost importance to consider the TME as a whole, rather than focusing solely on cancer cells. In the future, targeting other components of the TME, such as blood and lymph vessels, could become a viable strategy. A key ally in this effort is to unravel the epigenetic scenery of TME components and identify potential biomarkers that are TME-specific, distinguishing them from those in healthy cells. Moreover, newly emerged specific therapeutic methodologies or agents, such as multiomics and artificial intelligence (AI) technologies, nanoparticles (composite bioreactors, biomaterials-based nanoparticulate delivery systems), are expected to improve our efficacy in interrogating “druggability” of the lung TME epigenome 25-27. Finally, it is necessary to point out that the TME and epigenetic profiles vary not only between different tumor types but also among individual patients, emphasizing the critical need for precision medicine.

## Figures and Tables

**Figure 1:**
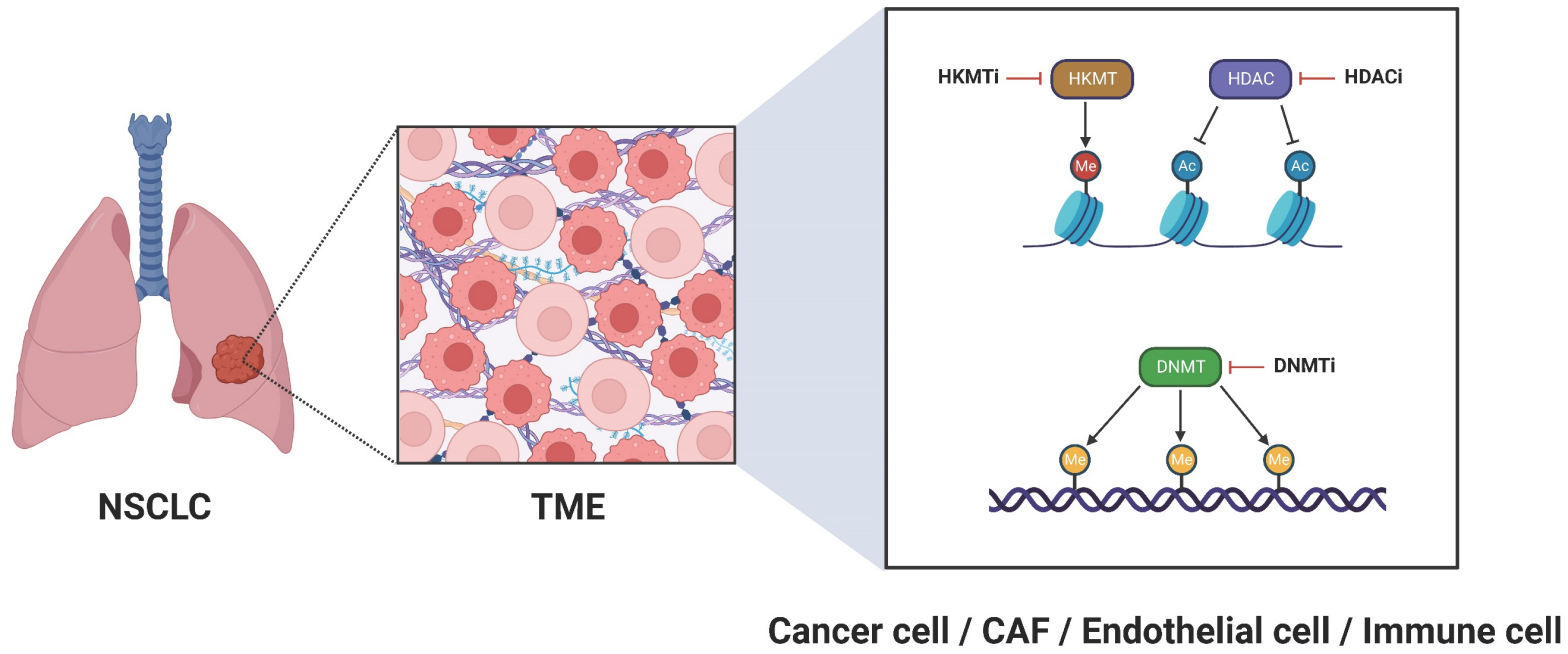
** Epigenetic targeting of the TME for NSCLC treatment.** Ac, histone acetylation; CAF, cancer-associated fibroblast; DNMT, DNA methyltransferase; DNMTi, DNA methyltransferase inhibitor; HDAC, histone deacetylase; HDACi, histone deacetylase inhibitor; HKMT, histone-lysine methyltransferase; HKMTi, histone-lysine methyltransferase inhibitor; Me (reddish circle), histone methylation; Me (yellowish brown circle), DNA methylation; NSCLC, non-small cell lung cancer; TME, tumor microenvironment.
